# Photoreceptor degeneration caused by defects of a ciliary kinase

**DOI:** 10.1186/2046-2530-1-S1-P96

**Published:** 2012-11-16

**Authors:** Y Omori, T Chaya, T Furukawa

**Affiliations:** 1 Osaka Bioscience Institute, Japan

## 

Defect of ciliary function causes photoreceptor degeneration in human ciliopathies including retinitis pigmentosa (RP), Leber's congenital amaurosis (LCA) and Bardet-Biedl syndrome (BBS). We show that a ciliary kinase, Mak, regulates retinal photoreceptor ciliary length and subcompartmentalization. Mak is localized both in the connecting cilia and outer segment axonemes of photoreceptor cells. In the Mak-null retina, photoreceptors exhibit elongated cilia and progressive degeneration. We observed accumulation of IFT88 and IFT57, expansion of Kif3a in the Mak-null photoreceptor cilia. In addition, overexpression of RP1, a microtubule-associated protein localized in outer segment axonemes, induced ciliary elongation, and Mak coexpression rescued excessive ciliary elongation by RP1. Our results suggest that Mak is essential for ciliary protein transport, regulation of ciliary length, acetylation of ciliary microtubules, and is required for the long-term survival of photoreceptors. We are currently studying roles of ciliary kinases in neuronal ciliogenesis.

